# DNA topology influences molecular machine lifetime in human serum†
†Electronic supplementary information (ESI) available: DNA sequences, fluorophore and quencher properties, equipment design, and degradation studies. See DOI: 10.1039/c5nr02283e



**DOI:** 10.1039/c5nr02283e

**Published:** 2015-05-11

**Authors:** Sara Goltry, Natalya Hallstrom, Tyler Clark, Wan Kuang, Jeunghoon Lee, Cheryl Jorcyk, William B. Knowlton, Bernard Yurke, William L. Hughes, Elton Graugnard

**Affiliations:** a Department of Materials Science & Engineering , Boise State University , Boise , Idaho 83725 , USA . Email: EltonGraugnard@BoiseState.edu ; Fax: +1-208-426-4466 ; Tel: +1-208-426-4026; b Department of Physics , Boise State University , Boise , Idaho 83725 , USA; c Department of Mathematics , Boise State University , Boise , Idaho 83725 , USA; d Department of Electrical & Computer Engineering , Boise State University , Boise , Idaho 83725 , USA; e Department of Chemistry & Biochemistry , Boise State University , Boise , Idaho 83725 , USA; f Department of Biological Sciences , Boise State University , Boise , Idaho 83725 , USA

## Abstract

DNA nanotechnology holds the potential for enabling new tools for biomedical engineering, including diagnosis, prognosis, and therapeutics. However, applications for DNA devices are thought to be limited by rapid enzymatic degradation in serum and blood. Here, we demonstrate that a key aspect of DNA nanotechnology—programmable molecular shape—plays a substantial role in device lifetimes. These results establish the ability to operate synthetic DNA devices in the presence of endogenous enzymes and challenge the textbook view of near instantaneous degradation.

## Abstract 2

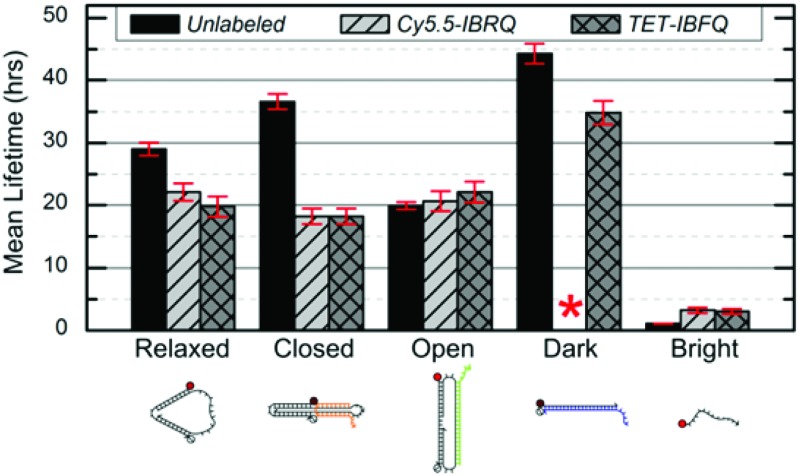
Lifetimes and operational performance were investigated for a DNA nanomachine and linear probe in human serum and blood to elucidate design principles for future biomedical applications of DNA-based devices.

## Introduction

Bio-inspired, rationally designed, DNA-based synthetic molecular machines have been constructed to perform work and a variety of functions based on changes in molecular conformation.^1–4^ With the aim of engineering programmable, synthetic macromolecules capable of mimicking and extending biological functions, these devices have been studied in buffers,^5,6^ animal sera,^6–8^ cellular environments,^8–19^ nematodes,^20–22^ cockroaches,^23^ and mice.^24–26^ However, little has been done to explore the viability of DNA devices in human blood and serum at physiologically relevant conditions.^15,27,28^ The performance and lifetime of DNA devices are thought to be greatly reduced by naturally occurring enzymes capable of degrading DNA within minutes^6,28–30^ – yet nuclease activity varies widely between species,^28,31^ and almost all testing of DNA devices in biological environments has been performed in solutions from non-human animals^6–12,20–26^ with relatively few studies in purely human-derived solutions.^14,15,27,28^

In this work, we performed a series of *in vitro* experiments designed to provide design rules for dynamic DNA nanomachines with the ultimate goal of developing new DNA-based tools for *in vivo* human biomedical diagnostic applications. To investigate topological influences on the lifetimes of DNA devices in biological fluids, we incubated a three-state DNA nanomachine (a.k.a. three-state DNA tweezers) and a two-state linear probe in human serum and fetal bovine serum. Experiments were performed at both 25 and 37 °C, corresponding to ambient and physiological temperatures, respectively. Degradation analysis revealed mean lifetimes of ∼20–36 hours for the three-state nanomachine and ∼44 hours for the double-stranded linear probe in human serum at 37 °C—roughly six times the mean lifetimes observed for the same devices in fetal bovine serum, underscoring the need for species-specific assays. Furthermore, device lifetimes varied greatly with topology (*i.e.*, circular *versus* linear) and molecular conformation (*i.e.*, shape of the structure), potentially providing a simple way to program viability or degradation. To assess the effects of enzymatic degradation on their dynamic function, we operated the three-state DNA nanomachine and the two-state linear probe in buffer, fetal bovine serum, and human serum, both at 25 and 37 °C. At 25 °C, both devices remained stable against enzymatic degradation with no enzymatic protection mechanisms required. At 37 °C, the devices exhibited signs of slow degradation in human serum consistent with their measured lifetimes. Additionally, we operated both devices in whole human blood at 25 °C, with operation virtually indistinguishable from that in buffer. Our results demonstrate the ability to operate DNA machines in the presence of endogenous enzymes and challenge the textbook view of instantaneous enzymatic degradation. Surprisingly, our data indicate that naked DNA device lifetimes are greater than devices with terminal fluorophore conjugation, in contrast to studies reporting enhanced nuclease resistance with extensive Cy-dye labeling or terminal chemical modifications.^7,28,29,32,33^ The potential for naked or minimally modified DNA device operation *in vivo* could enable regulation of enzymatic activity,^3^ control of transcription,^34^ and diagnostic imaging,^35^ greatly expanding the biomedical applications for DNA-based devices. Applications for operation in human blood range from non-viral gene and drug delivery^17,25,36–38^ to disease theranostics,^39–41^ and open the door for rationally designed protein analogs as new tools in biology, synthetic biology, and biotechnology.^35,42^

## Materials and methods

### DNA device design

**Fig. 1:**
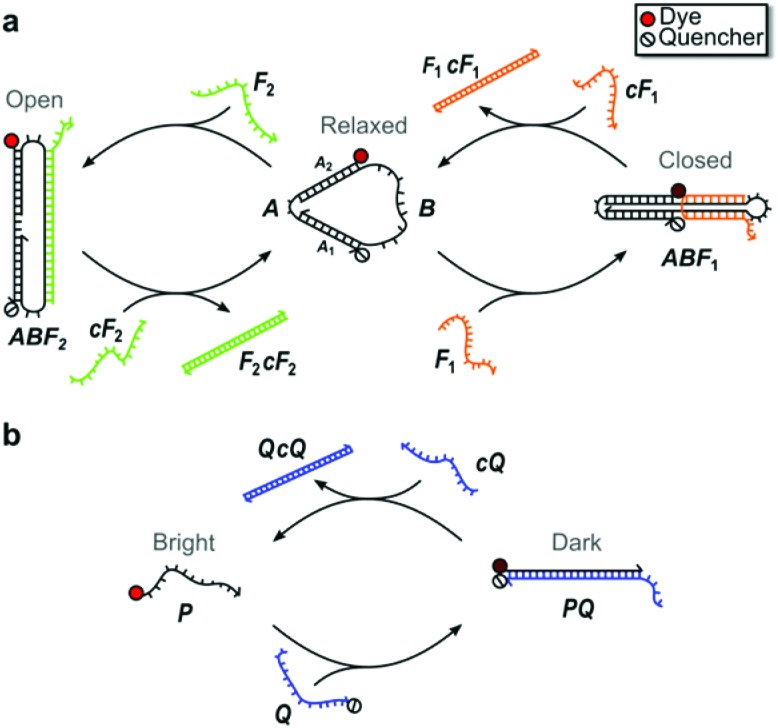
DNA nanomachine and linear probe schematics. (a) Three-state DNA nanomachine transitions between Relaxed, Closed, and Open states with the addition of fuel strands, *F*_1_ and *F*_2_, and their complements, cF_1_ and cF_2_. In the Relaxed state, the distance between the dye and quencher conjugated to double-stranded DNA segments A_1_ and A_2_ is estimated to be ∼5 nm. (b) The two-state linear probe transitions between the Bright and Dark states upon hybridization of the dye-labeled probe strand, P, and the quencher-labeled strand, Q. Strand displacement by cQ releases P and restores fluorescence emission. The mechanical states are monitored by measuring the fluorescence emission from the devices.

Fig. 1 illustrates the structure and operation of the nanomachine and linear probe, which form topologically distinct structures chosen to assess structurally-based differences in enzymatic degradation rates. The nanomachine used in this study (Fig. 1a) is a three-state device first reported by Simmel and Yurke^43^ as an extension of the two-state DNA tweezers.^1^ The nanomachine self-assembles into the Relaxed state and is switchable between its three distinct mechanical states (*i.e.*, Relaxed, Closed, Open) *via* hybridization with and strand displacement by complementary sets of DNA fuel strands acting on the single-stranded actuator domain of the nanomachine (labeled B in Fig. 1a).^1,43^ Details of nanomachine operation were reported previously,^43^ but briefly, fuel strand F_1_ closes the nanomachine by hybridizing with the actuator such that the two double-stranded DNA segments are bound together by a fuel cross-over. Complementary fuel, cF_1_, removes fuel F_1_ by toehold-mediated strand displacement,^1^ returning the nanomachine to its Relaxed state. Fuel F_2_ opens the nanomachine by hybridizing to the actuator so as to spread the double-stranded DNA segments. Fuel F_2_ is removed by strand displacement using its complement, cF_2_.

The proximity of an organic dye conjugated to one end of the double-stranded DNA segment A_1_ and a fluorescence quencher conjugated to the distal end of segment A_2_ allows the state of the nanomachine to be monitored by recording the fluorescence emission from the dye. The dye emission is modulated through fluorescence resonance energy transfer (FRET) to the quencher.^44^ This fluorescence emission was measured as a function of time as the nanomachine was repeatedly switched between all three states in order to visualize the kinetics of each state change. In the Relaxed state, the distance between the dye-quencher pair was previously estimated to be 5.1 nm^43^ and near the Förster distance for typical dye-quencher pairs, leading to an intermediate degree of quenching of the dye emission. In the Closed state, the dye-quencher pair separation is minimized by an adjacent cross-over of the fuel strand, yielding a separation of roughly 2 nm and leading to near complete emission quenching. In the Open state, the dye and quencher are separated by ∼13.6 nm, resulting in minimal dye quenching and maximum emission. Thus, the three-state DNA nanomachine is capable of multi-state reporting offering greater control and information for potential imaging and diagnostic advantages over binary two-state tweezers and beacons.^10^

Similar to a molecular beacon,^45^ the two-state linear probe, illustrated in Fig. 1b, consists of a set of complementary oligonucleotides, P and Q, conjugated to a fluorescent dye and adjacent quencher, respectively. In the double-stranded state, the fluorescence of the probe is quenched *via* FRET (Dark state). Instead of the hairpin recognition region of molecular beacons, the excess 10 nt single-stranded portion of the quencher strand serves as the toehold for hybridization with a 32 nt invasion strand (cQ) that is fully complementary to the quencher strand. After hybridization with the toehold, the linear probe strands undergo three-way branch migration, through which the invasion strand ultimately displaces the dye strand and restores emission of the probe (Bright state).

The sequences and domains for the nanomachine and linear probe are provided in ESI S1.† To enable observable operation in whole blood, which is highly absorbing below 600 nm, both devices were synthesized with the near-infrared dye Cy5.5 and IowaBlack RQ quencher (Cy5.5-IBRQ). Since a range of chemical DNA modifications are known to provide protection against enzymatic degradation,^7,28,29,32^ both the nanomachine and linear probe were also synthesized with the TET dye and IowaBlack FQ quencher (TET-IBFQ) and with no dye or quencher (unlabeled). The details of the dyes and quenchers and the absorption spectrum of heparinized whole human blood are provided in ESI S2.†

### Oligonucleotides and chemicals

DNA strands were purchased lyophilized from Integrated DNA Technologies with the purifications listed in Table S1.† The strands were rehydrated with filtered ultra-pure water and used without further purification. To synthesize the nanomachine (Fig. 1), strands A and B were combined in 1 × PBS (phosphate buffered saline, Sigma-Aldrich or Fisher Scientific), and the solution was annealed at 95 °C for five minutes and cooled to room temperature over ∼60 minutes. To prevent excess actuator in solution, strand A was added with 10% molar excess relative to B, which adds a slight offset to the fluorescence data. This solution was then added to 1 × PBS, heparinized whole blood, or serum to obtain the desired concentration and vortex mixed for 5 seconds.

### Sample purification

After synthesis and annealing, all samples for degradation studies were purified *via* native polyacrylamide gel electrophoresis (PAGE), with the exception of the Cy5.5-IBRQ labeled linear probe. As discussed in ESI S3,† a Cy5.5-IBRQ interaction led to agglomeration in PBS that impacted gel migration. Purification was performed using 1.5 mm thick native 10% polyacrylamide gels made in house with 1 × TAE, Mg^2+^ running buffer (40 mM tris, 20 mM acetic acid, 2 mM ethylenediaminetetracetic acid (EDTA), and 12.5 mM magnesium acetate; pH 8.0). Samples were loaded into the gels using 1 : 1 loading buffer of ficoll and 0.04 wt% bromophenol blue (both Sigma- Aldrich) and run at 150 V for 120 minutes at ∼20 °C. Bands of interest were cut from the gel, crushed, and eluted in 1 × PBS for >24 hours. Purified sample concentrations were calculated from absorbance measurements acquired with an Eppendorf Biophotometer Plus.

### Blood and serum collection

Whole human blood was collected from volunteers and prepared using standard protocols.^46^ Briefly, for whole blood experiments, blood was collected in heparin-coated vacutainers (BD, Fisher Scientific) to reduce clotting. Whole blood samples were stored at 4 °C for up to 21 days from the collection date. All whole blood experiments were performed using blood drawn from a single volunteer, and no difference in operation was observed in experiments run during the 21 day storage period. For serum experiments, blood was collected into silicone-coated serum vacutainers (BD, Fisher Scientific) and processed using standard protocols.^46^ Serum samples were frozen at –80 °C and stored for up to one year. All serum experiments were performed using serum collected from one volunteer, and a subset of the experiments was repeated in the serum of a second volunteer to confirm the results.

## Results and discussion

### Polyacrylamide gel electrophoresis degradation analysis

**Fig. 2:**
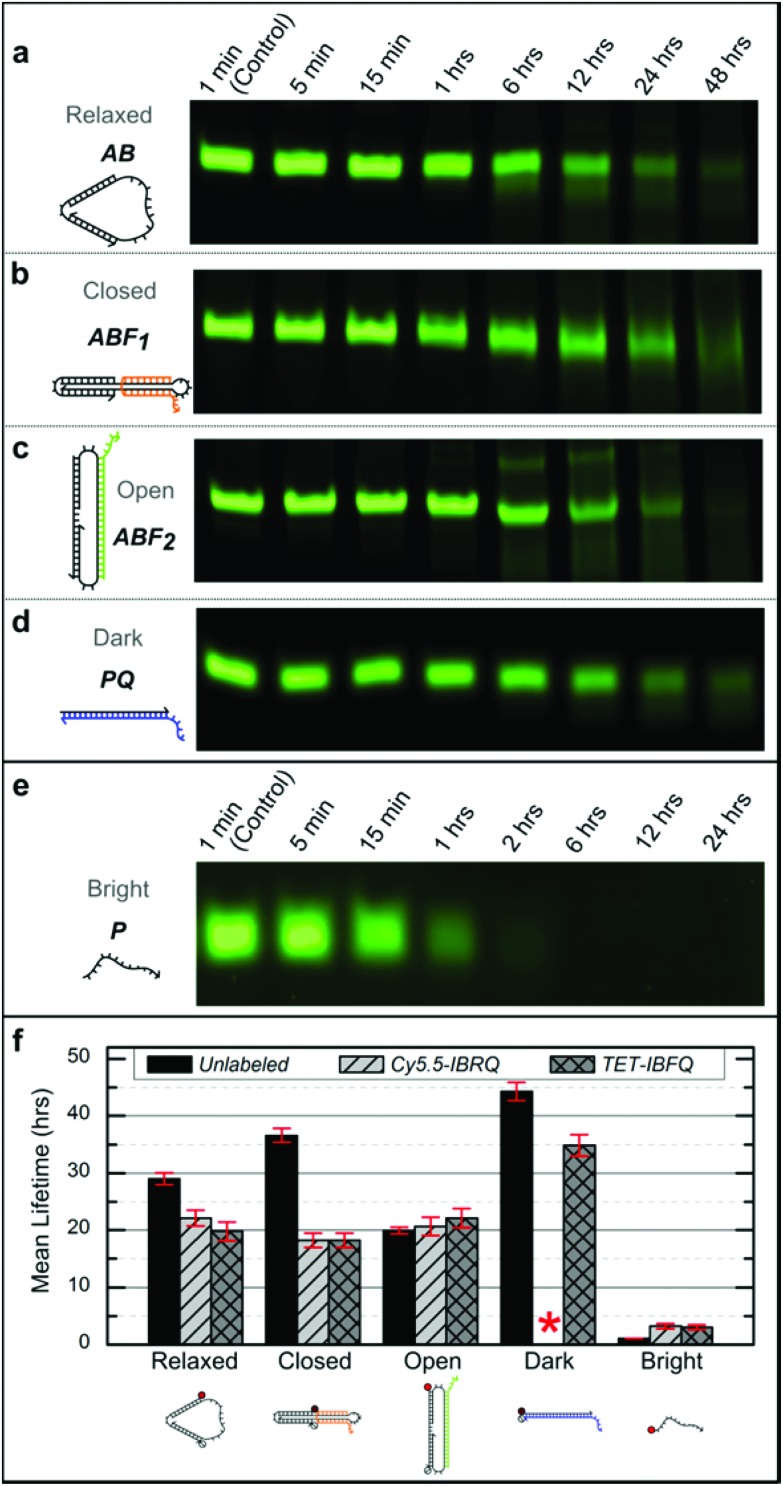
(a–e) Representative native 10% PAGE gel sections for the unlabeled nanomachine and linear probe after incubation in 70% human serum at 37 °C. Incubation times are indicated above each lane and were the same for the nanomachine states and the Dark linear probe. The incubation times for the Bright linear probe were shorter due to its considerably shorter lifetime. The nanomachine and Dark linear probe have bands visible even after 48 hours of incubation in human serum, while the Bright linear probe is completely degraded within 6 hours. For this figure, image contrast has been enhanced uniformly for individual gels to enhance the visibility of the faint bands. (f) Mean lifetimes (mean ± SE) from the DNA nanomachine and linear probe incubated in 70% human serum at 37 °C. Lifetimes were estimated based on normalized band intensities of native PAGE experiments, as described in ESI S4.† These data indicate considerable differences in degradation rates. No data is shown for the Cy5.5-IBRQ Dark linear probe (red asterisk) due to purification issues associated with Cy5.5-IBRQ interaction (see ESI S3†).

Support for long-term device viability in human serum was obtained through incubation of the nanomachine and linear probe in serum followed by native PAGE analysis to estimate device lifetimes. Samples were incubated for various periods in 70% human serum at 37 °C, corresponding to physiological temperature and where the activity of many DNA degrading enzymes is high,^47^ and then extracted using phenol : chloroform and analyzed *via* native PAGE (see ESI S4† for details). Fig. 2 shows representative PAGE data for the unlabeled (no dye or quencher) nanomachine and linear probe. Higher-order bands, such as those appearing in Fig. 2c, are thought to be the result of degrading dimers leftover from device purification prior to incubation. Lane profiles for unprocessed gel images were analyzed as described in ESI S4† and fit to a first-order exponential decay model to estimate the device mean lifetimes, which are shown in Fig. 2f. These data reveal significant differences in degradation rates that vary with molecular conformation and topology. The mean lifetime of the unlabeled Relaxed nanomachine in human serum was estimated to be 29.0 ± 1.0 hours (mean ± SE), while the Closed and Open nanomachine mean lifetimes were estimated to be 36.6 ± 1.2 and 19.9 ± 0.6 hours, respectively. The mean lifetime of the unlabeled Dark linear probe was estimated to be 44.3 ± 1.6 hours, while that of the Bright probe was estimated to be 1.1 ± 0.04 hours. As a confirmation of these results, both devices were incubated in human serum from a second volunteer, and the mean lifetimes of the Relaxed nanomachine and Dark probe were estimated to be ∼29 hours and ∼52 hours, respectively, which agree well with trends seen in results from the first volunteer. Mean lifetimes for the Cy5.5-IBRQ and TET-IBFQ modified devices are provided in Table S2.† These results indicate considerably longer lifetimes in human serum at physiologically relevant conditions than would be expected from typical lifetime assays in 10% non-heat-inactivated fetal bovine serum (FBS), where mean lifetimes on the order of minutes have been observed for structures similar in size to the devices studied here.^7,28^ Though nucleases are capable of degrading DNA within minutes under optimal digestion conditions,^6,29^ biological activity levels vary widely between species and are relatively low in humans.^31,48^ Fetal bovine serum, commonly used to supplement cell cultures and as a surrogate for *in vitro* biomedical studies,^6–10,28^ has high levels of nuclease activity and only mimics degradation conditions in human serum once it has been heat-inactivated.^28^ Impressive work has been done to enhance the stability of DNA structures and devices in hostile environments with lifetimes on the order of ∼40–200 hours even in 10% non-heat-inactivated FBS;^6,7^ however, such complex architectures and protection mechanisms may not be necessary for biomedical applications given the results demonstrated here.

For comparison with previous degradation studies,^6,7^ which use FBS, the unlabeled Relaxed nanomachine and Dark linear probe were incubated in non-heat-inactivated FBS at 37 °C for various periods and processed as described in ESI S4.† PAGE gel sections for FBS samples and representative PBS controls are shown in Fig. S3.† Based on incubation studies, the mean lifetime of the Relaxed nanomachine in FBS was estimated to be ∼4.4 hours, while that of the Dark linear probe was estimated to be ∼7.6 hours, roughly one-sixth of the lifetimes observed in human serum. These results further underscore the necessity of species-specific assays in assessing the potential biomedical applications of synthetic DNA devices.^22,28^

### Real-time device operation measurements in whole human blood and serum

While the PAGE data provide compelling evidence for long-term viability of DNA devices at physiological conditions, they do not assess the ability to dynamically operate DNA devices in biological environments. To assess the effect of enzymatic degradation on dynamic device function, the nanomachine and linear probe were operated with Cy5.5-IBRQ labels in human serum, whole human blood, and PBS at 25 and 37 °C, and their fluorescence emission was measured as a function of time. These DNA device reaction kinetics experiments were performed in 100% PBS; 97.5% human serum/2.5% PBS; and 97.5% heparinized whole human blood/2.5% PBS. Excitation and emission wavelengths and bandwidths are provided with the dye information in ESI S2,† while additional experimental details are provided in ESI S5 and S6.† A complete set of experiments was performed in serum and whole blood collected from one volunteer, and another subset was performed in serum from a second volunteer and in FBS. The human serum data sets were nearly identical, and kinetics data in human serum from the second volunteer and in FBS are provided in ESI S5.†

**Fig. 3:**
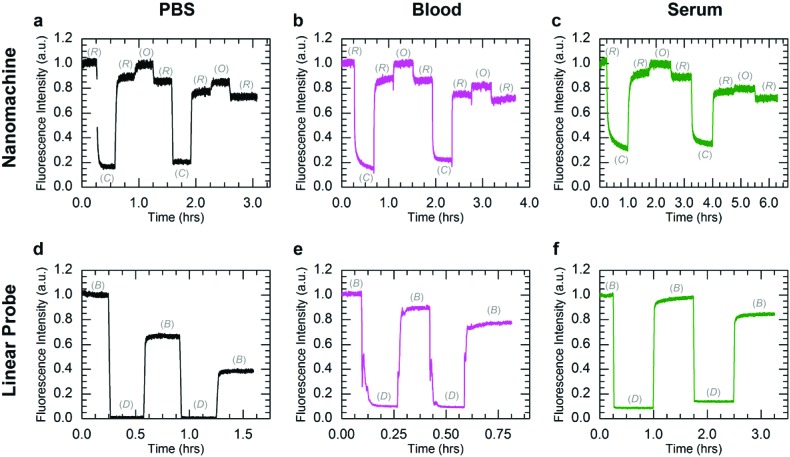
Kinetics data for operation of the three-state nanomachine and two-state linear probe. Both systems were operated in PBS (a, d), heparinized whole human blood (b, e), and human serum (c, f) through two complete cycles at 25 °C. The fluorescence data were normalized to the initial state intensities. The mechanical states of the nanomachine are Relaxed (R), Closed (C), and Open (O). The mechanical states of the linear probe are Bright (B) and Dark (D). Nanomachine data (a–c) include ∼10% fluorescence offsets from excess A in solution. Aside from the Cy5.5-IBRQ interaction in the linear probe in PBS (d, see main text), the observed overall decreases in fluorescence for the Relaxed state of the nanomachine and the Bright state of the linear probe are attributed to dilutions from fuel injections. Stable operation was observed for both systems in all three solutions.

Fig. 3 shows kinetics data for operation of both the nanomachine and linear probe at 25 °C at initial concentrations of 250 nM in PBS (Fig. 3a and d), heparinized whole human blood (Fig. 3b and e), and human serum (Fig. 3c and f). For this study, PBS was chosen as the buffer control since it is isotonic with whole blood and serum. State transitions were induced by injecting the appropriate fuel strand with a 50% molar excess at each stage. The high excess of fuel was added to drive the state transitions rapidly and decouple device degradation from fuel degradation. For each device, the kinetics data were normalized to the fluorescence intensities of the initial states, which were the Relaxed state for the nanomachine and Bright state for the linear probe. Operation was terminated arbitrarily after two complete cycles of state transitions, where one cycle was Relaxed-Closed-Relaxed-Open-Relaxed for the nanomachine, and Bright-Dark-Bright for the linear probe. The gradual decrease in Bright state fluorescence for the linear probe in PBS is attributed to Cy5.5-IBRQ interaction that inhibits strand dissociation in the linear probe, as described in ESI S3† and observed previously with similar dye-quencher modifications in cacodylate buffer.^49^ This Cy5.5-IBRQ interaction did not inhibit strand displacement in whole blood or serum, suggesting that the dye-quencher interaction is weaker in these media. For the nanomachine in PBS and both devices in whole blood and serum, the observed overall decrease in fluorescence for the Relaxed state of the nanomachine and the Bright state of the linear probe results from dilution from fuel injections. For the excitation intensities used in these experiments, evidence for photo-bleaching was not observed.^50,51^ The nanomachine and linear probe both exhibited surprisingly stable fluorescence emission and operated well in both heparinized whole human blood and human serum at room temperature over extended periods. While heparin does induce some inhibition of nuclease activity,^52^ operation in heparinized whole blood offers compelling support for the viability of active DNA machinery *in vivo*. Operation in human serum is even more compelling as enzymatic activity is higher, and both systems survived in human serum through two complete cycles of state transitions with no apparent indications of degradation.

**Fig. 4:**
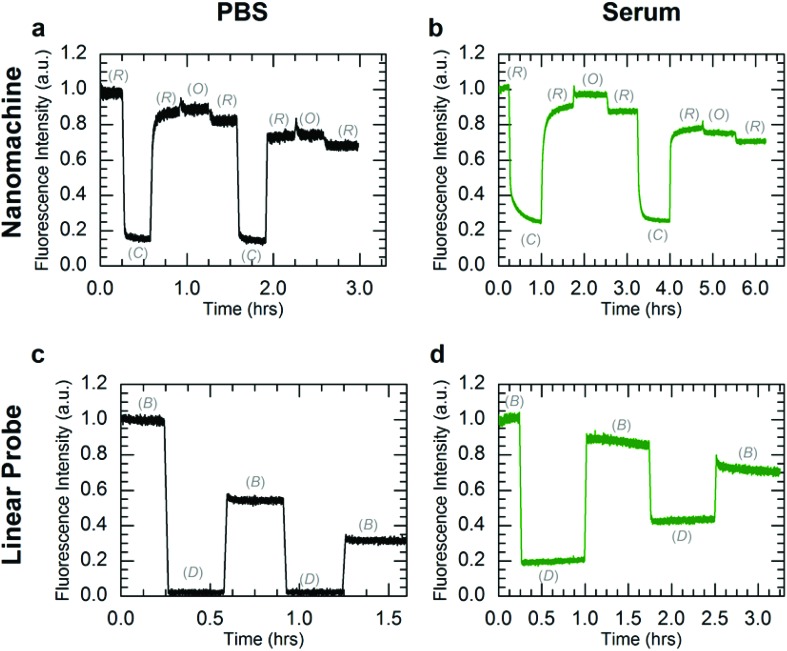
Kinetics data for operation of the three-state nanomachine and two-state linear probe at 37 °C. Both systems were operated in PBS (a, c) and human serum (b, d) through two complete cycles. The fluorescence data were normalized to the initial state intensities. The mechanical states of the nanomachine are Relaxed (R), Closed (C), and Open (O). The mechanical states of the linear probe are Bright (B) and Dark (D). Nanomachine data (a, b) include ∼10% fluorescence offsets from excess *A* in solution. The observed overall decreases in fluorescence for the Relaxed state of the nanomachine and the Bright state of the linear probe are attributed to dilutions from fuel injections. (a) Operation of the nanomachine in PBS shows state transitions similar to 25 °C, but with unstable fluorescence in the Open state. (b) In serum, clear state transitions were observed for the first complete cycle, yet the Open state transition failed during the second cycle. Operation was arbitrarily terminated after two complete cycles. (c) Operation of the linear probe in PBS is consistent with results seen at 25 °C. (d) In human serum, the linear probe displayed a continuous increase in Dark state fluorescence, which is attributed to enzymatic degradation.

To further investigate DNA nanomachine and linear probe viability for *in vivo* applications, both systems were operated in PBS and human serum at 37 °C. Instrument limitations did not allow for operation in whole human blood at physiological temperatures (see ESI S6†). The kinetics data for operation of the nanomachine and linear probe at 37 °C in PBS and human serum are shown in Fig. 4. The F_2_ fuel failed to hold the nanomachine open during both the first and second cycles, with the system displaying only a brief (∼5 minute) increase in Open-state fluorescence intensity before stabilizing at a lower intensity level. This instability of the Open state at 37 °C in PBS suggests a thermal instability (*i.e.*, unintended base pair dissociation) of the Open state in which the device is not able to maintain adequate separation of the dye-quencher pair. Thermal instability of this type can be mitigated by sequence optimization. Operation was terminated arbitrarily after two complete cycles of state transitions. In human serum at 37 °C (Fig. 4b), the nanomachine operates through 1.5 cycles nearly identically to operation in human serum at 25 °C (Fig. 3c). Similar to operation in PBS at 37 °C, a thermal instability of the Open state was also observed. As the two complete cycles were completed within six hours, well below the lifetimes of the various nanomachine states, degradation *via* nuclease activity was not discernible on these time scales. For comparison, clear evidence for nanomachine degradation was observed for operation in non-heat-inactivated FBS at 37 °C (Fig. S6a†).

Operation of the linear probe in PBS at 37 °C (Fig. 4c) is nearly identical to its operation at 25 °C (Fig. 3d). However, in contrast to the nanomachine, the linear probe displayed clear evidence for degradation in serum at 37 °C (Fig. 4d) with a decrease in the intensity difference between Bright and Dark states that is distinct from operation at 37 °C in PBS. It is likely that this degradation occurred primarily when the probe was in its single-stranded Bright state, which was shown to be much more vulnerable to nuclease activity in lifetime experiments. These results suggest enzymatic protection based on molecular conformation and topological differences between the circular nanomachine and the linear probe, even though both devices contain single and double-stranded domains. Further investigation is necessary to elucidate the topological nature of enzymatic resistance,^7,15,27^ which may play a role in enzymatic resistance of DNA origami devices and nanostructures.^6,7,14^

For further comparison with previous degradation studies,^6,7^ the Cy5.5-IBRQ nanomachine was also operated at 25 and 37 °C in non-heat-inactivated FBS. The kinetics data for operation in FBS are shown in Fig. S6a† and show clear evidence for degradation at a considerably greater rate than in human serum. The kinetics data for operation in the serum from a second volunteer are shown in Fig. S6c.† These results indicate short-term viability of the nanomachine in FBS, but confirm the need for species-specific assays^22,28^ and for further study of the relevance of non-heat-inactivated FBS assays for potential *in vivo* applications for humans.^14,15,27,28^

One must be cautious even when comparing studies in serum from the same species, as experimental dilution of sera can have dramatic effects on nuclease activity. Hahn *et al.* found dilution of FBS in low-magnesium media to greatly decrease nuclease activity, though this effect was countered by supplementing with millimolar concentrations of MgSO_4_.^8^ While still useful, studies performed in FBS diluted in media that have not been supplemented with magnesium will therefore not be directly comparable due to the change in nuclease activity.^7^ In human serum, DNaseI activity is naturally inhibited by actin and saline,^48,53^ and studies performed in diluted human serum run the risk of reversing this inhibition and artificially increasing nuclease activity by diluting the inhibitors.^28,48^

When designing *in vitro* experiments, it is necessary to choose experimental conditions that best reflect the *in vivo* biological conditions of interest. In the lifetime studies reported here, we have attempted to do that by using minimal dilution of sera in PBS, which should not substantially reverse inhibition of nuclease activity in human serum samples because saline concentration is maintained. Samples for lifetime studies in sera were diluted to a 7 : 3 mixture of serum and PBS, which was limited by the concentration of DNA samples in PBS following PAGE purification. Samples for kinetics measurements were not purified prior to operation and were therefore able to be kept at a higher serum percentage while maintaining the same DNA device concentration. Though our experimental conditions should not have reversed naturally occurring nuclease inhibition, it is possible the slight decrease in magnesium concentration did lead to a proportional decrease in nuclease activity, particularly for 10% FBS dilutions used in comparative kinetics experiments (Fig. S6a†). Nonetheless, the results reported here for nanomachine and linear probe lifetimes agree well with the most comparable studies in human serum: Chu and Orgel reported substantial degradation of single-stranded DNA in 90% human serum within 2 h, and nearly complete degradation occurring within 4 h.^15^ They also reported that double-stranded and hairpin structures remained largely intact, even after overnight incubation. Furthermore, they found that ligated dumbbell DNA remained the most stable, with degradation eventually occurring *via* endonuclease nicks of the single-stranded dumbbell regions and subsequent exonuclease digestion. Di Giusto and King also found ligated structures to be extremely robust, with half-lives of 6.9–13.6 h.^27^ It is not clear what serum percentages were used for those assays, and differences in dilution conditions may account for the shorter lifetimes relative to those reported here. Di Giusto and King noted that even unligated circular structures exhibited increased lifetimes, possibly a result of decreased availability of DNA termini to exonuclease digestion. Despite the presence of both single- and double-stranded regions, the nanomachine exhibits mean lifetimes between 18 and 36 h for all conformation and dye-quencher variations, likely a result of its circular topology.^15,27^ In contrast to previous studies, our results show that the Dark linear probe exhibits the longest mean lifetime, despite the fact that it contains only single- and double-stranded DNA with no hairpins or circular constructs. The nature of this discrepancy requires further study; however, the dynamic functionality of the probe will be much less than the lifetimes reported here, since transitioning to the Bright state greatly increases the device's degradation rate, as shown in Fig. 2e and 4d.

To assess the effect that partial digestion of the actuator would have on nanomachine operation and PAGE results, the nanomachine was synthesized with a single nick in the middle of the actuator, similar to the original DNA tweezer^1^ (details in ESI S7†). A single nick of the actuator does not significantly impact nanomachine operation, though the ability to monitor its operation—particularly the transition between the Relaxed an Open states—*via* fluorescence spectroscopy is diminished, as shown in Fig. S9b.† Differences in the PAGE migration rates for nicked structures were significant for the Closed nanomachine, but the migration rates of Relaxed and Open structures were unaffected by the presence of a nick on the actuator (Fig. S10†); thus, the mean lifetimes of the Relaxed and Open states reported here may include minimally digested devices.

Interestingly, the Cy5.5-IBRQ and TET-IBFQ labeled nanomachine and linear probe displayed shorter mean lifetimes than their unlabeled counterparts for all but the Open nanomachine, in contrast to previous studies showing enhanced enzymatic resistance with extensive Cy-dye labeling or terminal chemical modifications.^7,28,29,32,33^ Moreira *et al.* investigated the effect of fluorophores and quenchers on the thermodynamic stability of duplex DNA.^49^ They reported increased duplex stability in cacodylate buffer for many dye-quencher modifications, including the Cy5-IBRQ pair. Cy5 is very similar in structure to the Cy5.5 dye used in the studies reported here, suggesting a comparable phenomenon may be responsible for the apparent inhibition of strand dissociation for the Cy5.5-IBRQ linear probe in PBS (Fig. 3d and 4c). This stabilizing effect appears to be dependent on the reaction buffer composition, as demonstrated by uninhibited strand displacement for the Cy5.5-IBRQ linear probe in human serum and whole blood at 25 °C (Fig. 3e and f). Moreira *et al.* also found some dye-quencher modifications, including both TET and IBFQ, to have a slightly adverse effect on duplex stability. It is possible that in serum, both Cy5.5-IBRQ and TET-IBFQ lead to decreased duplex stability, increasing breathing and thereby making the DNA strands more susceptible to enzymatic attack; however, this effect was not uniform across all three nanomachine states, with the Open nanomachine displaying approximately equal lifetimes for all three dye-quencher variations. These discrepancies may be due to the differences in molecular conformation of the three nanomachine states, with the Open state leaving the termini of segments A_1_ and A_2_ more exposed than in the Relaxed or Closed states, thereby increasing susceptibility to exonuclease attack and overshadowing dye-quencher effects.

## Conclusion

DNA-based devices offer interesting possibilities for programmable gene therapy, drug delivery and diagnostics; however, little was previously known about how such devices operate in human blood and serum.^15,27,28^ The devices reported here are active, programmable, synthetic machines capable of surviving in human serum under physiological conditions roughly six times longer than would be predicted by FBS studies. While studies in sera from non-human animals are important, the higher level of nuclease activity relative to human serum in commonly used media, such as FBS, underestimates the viability of DNA-based devices for human biomedical applications.^28^ Our results challenge the textbook notion of nearly instantaneous degradation of DNA devices in biological environments and demonstrate the ability to dynamically operate nucleic acid machinery in human blood and serum in the presence of endogenous enzymes. These findings offer potential tools for *in vitro* applications, particularly at room temperature where DNA device lifetime is even greater than the 37 °C values reported here, with 25 °C kinetics measurements revealing operation equivalent to buffer. The dependence of degradation rate on both molecular conformation and topology also provide a means of intentionally programming digestion rates for particular applications.

More significantly, viable operation at physiological conditions has important implications for *in vivo* applications, especially considering the long lifetime (∼20–36 h mean lifetime for the three nanomachine states) of unmodified/unlabeled DNA devices. Although labeling is required for many imaging applications, programmable drug delivery and gene therapy can be adversely affected by conjugated molecules that can reduce specificity and/or operability. Thus, extensive modifications and protection mechanisms that may inhibit the intended operation, reduce target specificity (*e.g.*, the PEG problem^36^), and add additional cost may, in fact, not be necessary. This has positive implications for the operation of naked and minimally-modified DNA-based molecular machines and computational systems *in vivo* in humans. On the other hand, even the dye/quencher labeled DNA-based devices exhibit functionally useful lifetimes (>18 h for all but the single-stranded Bright probe), suggesting use in dynamic imaging applications where programmable machine operation can provide more nuanced diagnostic information than simple on/off signaling. Additionally, although degraded considerably faster than in human serum, short-term viability in non-heat-inactivated FBS was also demonstrated. Further studies are necessary to validate the topological enzyme resistance exhibited by the nanomachine and similar circularized DNA structures,^7,15,27^ to assess the effectiveness of this resistance in circulation,^30^ and to determine the time scales on which these devices would be cleared by the kidneys relative to their lifetimes.^24,30^ Such work will open the door to allowing programmable functions, including genetic regulation, to be performed within humans.
