# Supplementary material for: DNA topology influences molecular machine lifetime in human serum
Source: Nanoscale. 2015 May 11;7(23):10382–90. doi: 10.1039/c5nr02283e (PMC4457601; doi:10.1039/c5nr02283e)
Supplement: Supplementary file 1 [file NR-007-C5NR02283E-s001.pdf]

Electronic Supplementary Material (ESI) for Nanoscale.
This journal is © The Royal Society of Chemistry 2015

(Electronic Supplementary Information)

DNA Topology Influences Molecular Machine Lifetime in Human Serum

Sara Goltry,[a] Natalya Hallstrom,[a] Tyler Clark,[b,c] Wan Kuang,[d] Jeunghoon Lee,[a,e] Cheryl Jorcyk,[f] William B. Knowlton,[a,d] Bernard Yurke,[a,d] William L. Hughes[a] and Elton Graugnard*[a]

[a] *Department of Materials Science & Engineering*
[b] *Department of Physics*
[c] *Department of Mathematics*
[d] *Department of Electrical & Computer Engineering*
[e] *Department of Chemistry & Biochemistry*
[f] *Department of Biological Sciences*
Boise State University, Boise, Idaho 83725, USA
** To whom correspondence should be addressed. Tel: 1-208-426-4026; Fax: 1-208-426-4466; Email: EltonGraugnard@BoiseState.edu*

Table of Contents: Supplementary Information **Page #**

Section	Page #
**S1: DNA Nanomachine and Linear Probe Domains and Sequences**	2
**S2: Dye and Quencher Details, Absorption Spectrum of Whole Blood**	3
**S3: Cy5.5-IBRQ Interaction**	4
**S4: Incubation in Serum followed by Native PAGE Analysis**	5
**S5: Kinetics experiments in Buffer, Human Serum, Blood, and FBS**	8
**S6: Custom Laser-Based Epi-Fluorescence Photometer**	10
**S7: Kinetics and Native PAGE Results for Nicked Nanomachine in Buffer**	11

Supplementary Information S1: DNA Nanomachine and Linear Probe Domains and Sequences

As illustrated in Fig. 1, the DNA nanomachine consists of two stiff double-stranded DNA segments (A_1, A_2), each 18 base-pairs long and connected by a flexible four nucleotide (nt) single-stranded DNA hinge and a flexible 48 nt long single-stranded DNA actuator (*B*). The two-state linear probe consists of a 22 nt strand (*P*) conjugated to a fluorescent dye at its 5' end and a 32 nt strand (*Q*) conjugated to a fluorescence quencher at its 3' end. The domains and sequences for the nanomachine and linear probe are given in Table S1. For clarity, functional domains are segmented (-) at toeholds and colored by complementarity. Note that fuel complements are not listed, and the colors in Table S1 are independent of Fig. 1. The dye-quencher pairs were Cy5.5-IBRQ or TET-IBFQ. Both systems were also synthesized with no dye or quencher.

TABLE S1. DNA Nanomachine and Linear Probe Domains and Sequences.

Strand Name	Sequence (5' to 3')	Length (nt)	Purification
**DNA Nanomachine (same as Ref. 1)**			
A	/Dye/TGCCTTGTAAGAGCGACCATCAACCTGGAATGCTTCGGAT/ Quencher/	40	HPLC *
B	GGTCGCTCTTACAAGGCACTGGTAACAATCACGGTCTATGCGGG AGTCCTACTGTCTGAACTAACGATCCGAAGCATTCCAGGT	84	PAGE **
F_1	CCGTGATTGTTACCAGCGTTAGTTCAGA-TGCTACGA	36	HPLC
F_2	AGTTCAGACAGTAGGACTCCCGCATAGACCGTGATTGTTA- TGCTACGA	48	HPLC
**Linear Probe**			
P	/Dye/AAACCGTTACCATTACTGAGTT	22	HPLC
Q	GTAGTGTGGG-AACTCAGTAATGGTAACGGTTT/Quencher/	32	HPLC

* High-purity liquid chromatography
** Polyacrylamide gel electrophoresis

Supplementary Information S2: Dye and Quencher Details, Absorption Spectrum of Whole Blood

The common names for the dyes used in this study are

- Cy5.5™: 1-[3-(4-monomethoxytrityloxy)propyl]-1'-[3-[(2-cyanoethyl)-(N,N-diisopropyl)phosphoramidityl]propyl]-3,3,3',3'-tetramethyl-4,5-benzindodicarbocyanine iodide. Molecular weight: 1171.25. For serum studies in an Agilent Technologies Cary Eclipse fluorescence spectrophotometer, Cy5.5 was excited at 685 ± 1.25 nm, and its emission monitored at 706 ± 5 nm. For whole blood studies in a custom epi-fluorescence system (described below), Cy5.5 was excited with a laser diode operating at 670 nm and reflected to the sample by a dichroic mirror. Emission above 710 nm was passed through the dichroic mirror to the detector (see Supplementary Information S6 for details).
- TET™: 6-(4, 7, 2', 7'-Tetrachloro-3', 6'-dipivaloylfluoresceinyl-6-carboxamido)-hexyl-1-O-(2-cyanoethyl)-(N,N-diisopropyl)-phosphoramidite. Molecular weight: 981.73. For serum studies in an Agilent Technologies Cary Eclipse fluorescence spectrophotometer, TET was excited at 510 ± 1.25 nm, and its emission monitored at 538 ± 5 nm.
- IBRQ: IowaBlack® RQ, no additional chemical information is available. IBRQ has a peak absorbance at 656 nm.
- IBFQ: IowaBlack® FQ, no additional chemical information is available. IBFQ has a peak absorbance at 531 nm.

The absorption spectrum of a 1% solution of whole human blood in 1×PBS was measured using an Agilent Technologies Cary 300 and is shown in Fig. S1. The excitation and emission wavelengths are indicated for the TET and Cy5.5 dyes. The bar widths correspond to the instrument bandwidths used for data acquisition.

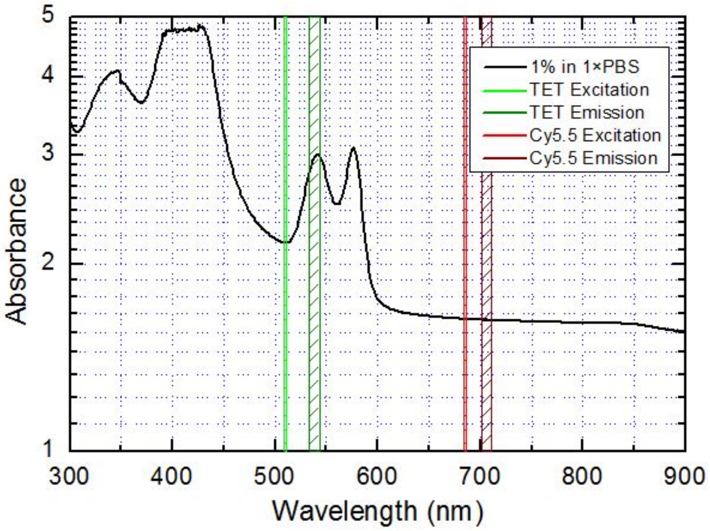

Fig. S1. Absorption spectrum for a 1% solution of whole human blood in 1×PBS. For reference, the excitation and emission wavelengths and bandwidths for the TET and Cy5.5 dyes are indicated by the vertical hatched regions.

Supplementary Information S3: Cy5.5-IBRQ Interaction

Prior to incubation in 70% serum for native polyacrylamide gel electrophoresis (PAGE) experiments (described in Supplementary Information S4), the nanomachine and linear probe were purified via native PAGE to filter out excess DNA and malformed structures. The Cy5.5-IBRQ linear probe appeared to agglomerate in solution and could not be purified. Neither the Cy5.5-IBRQ nanomachine nor the TET-IBFQ devices exhibited this behavior. Fig. S2 shows the results of a typical native PAGE purification attempt with multiple variations of the dye-quencher combinations on the linear probe. As seen in the gel image, both Cy5.5-IBRQ and Cy5.5-IBFQ linear probe exhibited the agglomeration and slow migration behavior. Moreira, et al. found various dye-quencher modifications to alter the thermodynamic stability of duplex DNA.[1] Though they did not specifically study the Cy5.5-IBRQ pair, they did find that similar cyanine dyes and IBRQ increased duplex stability in cacodylate buffer. It is possible that this phenomenon is partially to blame for the strange gel migration behavior of our probe, though the nature of the Cy5.5-quencher interaction requires further study.

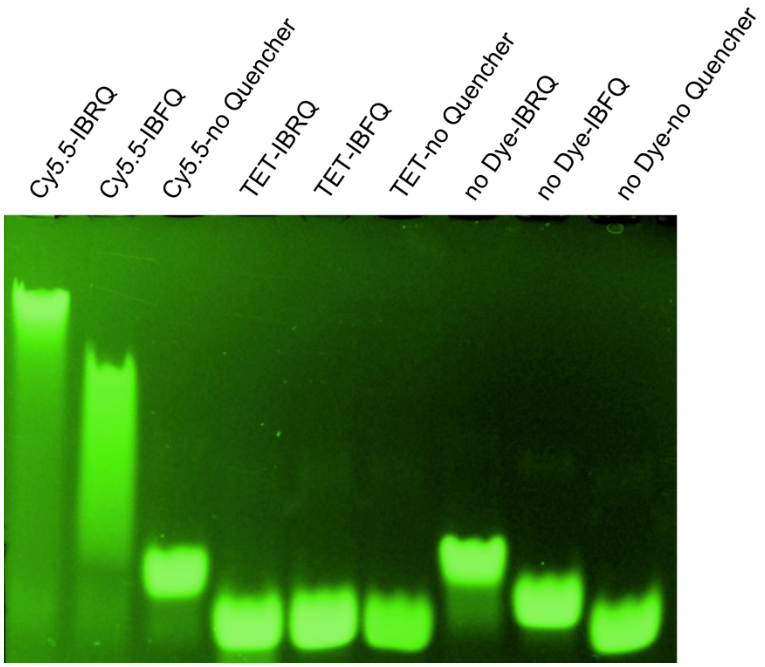

Fig. S2. Native PAGE gel for attempted purification of the linear probe. Dye-quencher modifications are indicated above each lane. The gel was run at 20 °C at 150 V for 120 minutes. The Cy5.5-IBRQ and Cy5.5-IBFQ probes in the two left lanes appear to agglomerate and migrate at a very slow rate. All other dye-quencher permutations of the Dark probe migrated at similar rates, as shown in the seven other lanes.

Supplementary Information S4: Incubation in Serum followed by Native PAGE Analysis

Samples for degradation experiments were prepared at 250 nM in a 100 µL 7:3 serum:PBS mixture and incubated at 37 °C for varying times. Diluting human serum can shorten DNA device lifetimes, as endogenous levels of actin and saline in human serum greatly inhibit the activity of some nucleases.[2] Note that dilution with PBS, as done here, should not reverse the inhibition of nuclease activity in human serum. After incubation, the DNA from the samples was extracted using 50 µL chloroform and 50 µL 25:24:1 phenol:chloroform:isoamyl alcohol (Sigma-Aldrich), followed by centrifugation at 13,000 rcf for 10 minutes. PAGE analysis of the degraded samples was performed using 1.5 mm thick native 10% polyacrylamide gels made with 1×TAE, 6.25 mM magnesium acetate, pH 8.0 gel and running buffer. Samples were loaded using 19:1 loading buffer of ficoll and 0.04 wt% bromophenol blue and run at 150 V for 120 minutes at 20 °C. Completed gels were stained with SYBR Gold (Life Technologies) and imaged using a multiplexed fluorescence detection and gel documentation system (FluorChemQ, ProteinSimple). Representative native PAGE gel sections for human serum samples are shown in Fig. 2, while gel sections for non-heat-inactivated fetal bovine serum (FBS) samples and representative PBS controls are shown in Fig. S3.

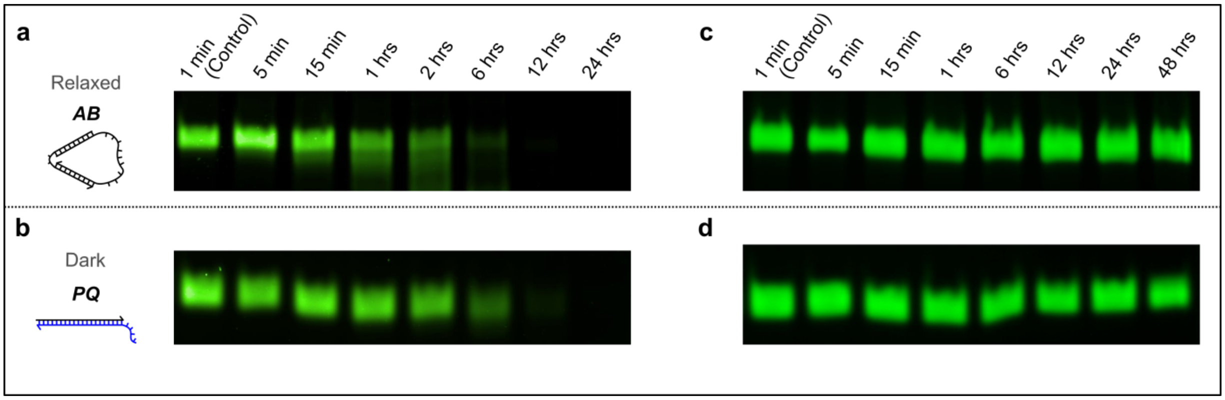

Fig. S3. Representative native 10% PAGE gel sections for the unlabeled (a) nanomachine and (b) linear probe after incubation in 70% non-heat-inactivated FBS at 37 °C. Corresponding controls for incubation in 1×PBS at 37 °C are shown in (c) and (d), respectively. Incubation times are indicated above each pair of gels.

To quantify the degree of degradation, intensity profiles were acquired from each incubation time stamp lane. The background intensity of each lane was subtracted from the intensity profile to obtain the net peak height prior to normalization, as illustrated in Fig. S4. The net peak heights of all samples for a particular gel were then normalized to the net peak height of the 1 min incubation sample from that gel. This normalized intensity reflects an approximate fraction of operational devices remaining at a particular time. Fig. S5 shows the normalized intensity-time profiles. After baseline subtraction, but prior to normalization, band intensities for the first two time stamps were frequently approximately equal, with variations primarily due to pipetting and

experimental error, indicating that very little degradation occurred in the first few minutes of incubation and validating the choice of the 1 min incubation sample as the control for normalization.

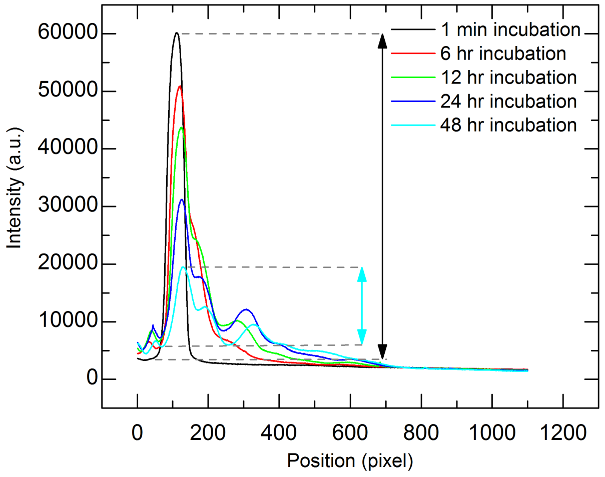

Fig. S4. Representative native PAGE band profiles from selected time stamps for the gel section shown in Fig. 2a. The background intensity from each lane was subtracted from the peak intensity to determine the net peak height for each time stamp. For example, the net peak heights of the 1 minute and 48 hour incubation lanes are indicated by double-headed arrows.

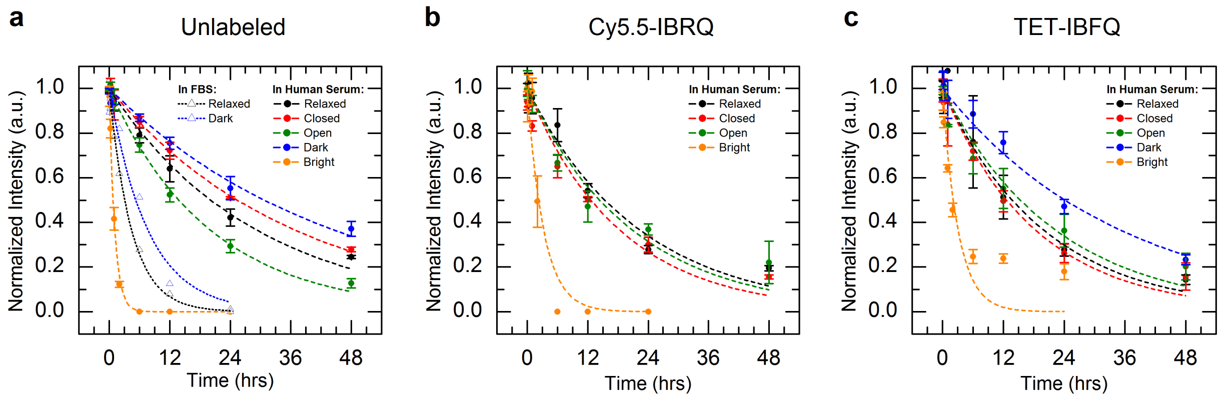

Fig. S5. Normalized band intensity-time profiles for the (a) unlabeled, (b) Cy5.5-IBRQ, and (c) TET-IBFQ nanomachine and linear probe after incubation in 70% human serum at 37 °C. Average and standard deviations for n=3 trials of each time stamp are shown for incubation in human serum. For comparison, data for n=1 trial are also shown after device incubation in 70% non-heat-inactivated FBS in (a). Dashed curves show first-order exponential decay fits. No data is shown for the Cy5.5-IBRQ Dark probe in (b) because of purification issues, as described in Supplementary Information S3.

As in previous studies,[3,4] the data were fit to a first-order exponential decay model:

$$[\mathrm{D}] = [\mathrm{D}]_0 e^{-\lambda \cdot t} \qquad \text{(S1)}$$

where [D] is the DNA device concentration, $[\mathrm{D}]_0$ is the initial concentration, t is the incubation time, and λ is the decay rate.

The decay rate is related to the mean lifetime τ by

$$\lambda = \frac{1}{\tau}. \tag{S2}$$

The half-life $t_{1/2}$ can then be calculated as

$$t_{1/2} = \tau \cdot \ln 2. \tag{S3}$$

Substituting Eq. (S2) into (S1) and dividing by $[D]_0$ to get an expression for the normalized band intensity I yields

$$I = \frac{[D]}{[D]_0} = e^{-t/\tau}. \tag{S4}$$

The data were fit to Eq. (S4) using OriginPro software to obtain values for τ, which were then used to calculate $t_{1/2}$ and λ. The results of this analysis are shown graphically as dashed curves in Fig. S5 and numerically in Table S2. Mean lifetimes in human serum are also shown in Fig. 2f.

Nearly all of the degradation data were well described by the first-order exponential decay model ($R^2 > \sim 0.93$), but it was noted that the TET-modified Bright probe appeared to settle out to a non-zero final fluorescence, indicating possible failure of this model (Fig. S5c, orange data set, $R^2 = \sim 0.86$). Similar behavior was observed, though not discussed, in data from previous publications (Conway, et al., Supplementary Figure S12, $T80_{HEG}$ data set) and may warrant modification of the mathematical model used for such degradation studies.[4]

TABLE S2. Results of first-order exponential decay analysis for the DNA nanomachine and linear probe incubated in 70% human serum and 70% non-heat-inactivated FBS at 37 °C (fitted curves shown in Fig. S5). No data is given for the Cy5.5-IBRQ Dark probe because of purification issues, as described in Supplementary Information S3.

**Incubation Media**	**Device State**	**Modification**	**# of trials**	**τ ± SE (hrs)**	**$t_{1/2}$ ± SE (hrs)**	**λ ± SE (hrs^{-1})**	**R^2**
**Human Serum**	Relaxed	Unlabeled	3	29.0± 1.0	20.1 ± 0.7	0.0345 ± 0.0012	0.98312
		Cy5.5-IBRQ	3	22.2 ± 1.4	15.4 ± 1.0	0.0451 ± 0.0028	0.96215
		TET-IBFQ	3	19.8 ± 1.7	13.7 ± 1.2	0.0505 ± 0.0043	0.94050
	Closed	Unlabeled	3	36.6 ± 1.2	25.3 ± 0.8	0.0274 ± 0.0009	0.98446
		Cy5.5-IBRQ	3	18.2 ± 1.3	12.6 ± 0.9	0.0549 ± 0.0038	0.94844
		TET-IBFQ	3	18.2 ± 1.3	12.6 ± 0.9	0.0550 ± 0.0038	0.95344
	Open	Unlabeled	3	19.9 ± 0.6	13.8 ± 0.4	0.0503 ± 0.0015	0.99239
		Cy5.5-IBRQ	3	20.7 ± 1.6	14.3 ± 1.1	0.0484 ± 0.0038	0.93454
		TET-IBFQ	3	22.1 ± 1.7	15.3 ± 1.2	0.0452 ± 0.0034	0.93233
	Dark	Unlabeled	3	44.3 ± 1.6	30.7 ± 1.1	0.0226 ± 0.0008	0.9775
		Cy5.5-IBRQ	n/a	n/a	n/a	n/a	n/a
		TET-IBFQ	3	34.8 ± 1.9	24.1 ± 1.3	0.0287 ± 0.0016	0.96499
	Bright	Unlabeled	3	1.10 ± 0.04	0.76 ± 0.03	0.9102 ± 0.0338	0.99418
		Cy5.5	3	3.24 ± 0.46	2.25 ± 0.32	0.3087 ± 0.0436	0.92899
		TET	3	3.06 ± 0.42	2.12 ± 0.29	0.3270 ± 0.0446	0.85974
**FBS**	Relaxed	Unlabeled	1	4.4 ± 0.2	3.1 ± 0.2	0.2256 ± 0.0119	0.99561
	Dark	Unlabeled	1	7.6 ± 0.8	5.3 ± 0.6	0.1312 ± 0.0141	0.97713

Supplementary Information S5: Kinetics experiments in Buffer, Human Serum, Blood, and FBS

To assess the effect of enzymatic degradation on dynamic device function, the nanomachine and linear probe were operated with Cy5.5-IBRQ labels in human serum, whole human blood, and PBS at 25 and 37 °C, and their fluorescence emission was measured as a function of time, as shown in Figs. 3 and 4. These DNA reaction kinetics experiments were performed in 100% PBS; 97.5% human serum/2.5% PBS; and 97.5% heparinized whole human blood/2.5% PBS. Fuels were added in constant volume and varying concentration so that each injection was at 50% molar excess relative to the previous injection. The high excess of fuel was added to drive the state transitions rapidly and decouple device degradation from fuel degradation. Teflon coated magnetic stir bars (Fisher Scientific) were used to mix solutions or solutions were mixed by hand using pipettes. Experiments were run in 4 mm specialty optical glass cuvettes (Starna Cells) or 10 mm methacrylate cuvettes (Fisher Scientific).

Kinetics measurements in PBS and serum were acquired using Agilent Technologies Cary Eclipse fluorescence spectrophotometers using the excitation and emission wavelengths provided in Supplementary Information S2. Due to the high absorbance of blood and the 90° orientation of the excitation and emission paths within Cary Eclipse spectrophotometers, kinetics measurements in whole blood were recorded using a custom laser-based fluorescence photometer designed for Cy5.5, as described in Supplementary Information S6.

The nanomachine was also operated in fetal bovine serum (FBS) and the serum from a second volunteer. In FBS, the nanomachine kinetics data exhibited clear evidence for degradation. As seen in Fig. S6a, the fluorescence of the Closed state reached only 50% of the Relaxed state intensity and returned to only 60% in the second Relaxed state. However, the large increase in intensity when transitioned to the Open state indicates that the primary degradation was to the exposed single-stranded toehold region of the F_1 fuel strand. As seen in the schematics (Fig. 1a), the fuel F_2 can Open the Closed nanomachine by binding directly to the available single-stranded portion of the actuator and displace any F_1 fuel strands that were rendered inaccessible to the cF_1 strand by degradation of the toehold. Nonetheless, after 200 minutes, the nanomachine was rendered inoperable and state transitions for the second cycle were greatly diminished. The kinetics data in serum from a second volunteer (Fig. S6c) were virtually indistinguishable from those in the serum of the first volunteer (Fig. S6b, reproduced from Fig. 4b).

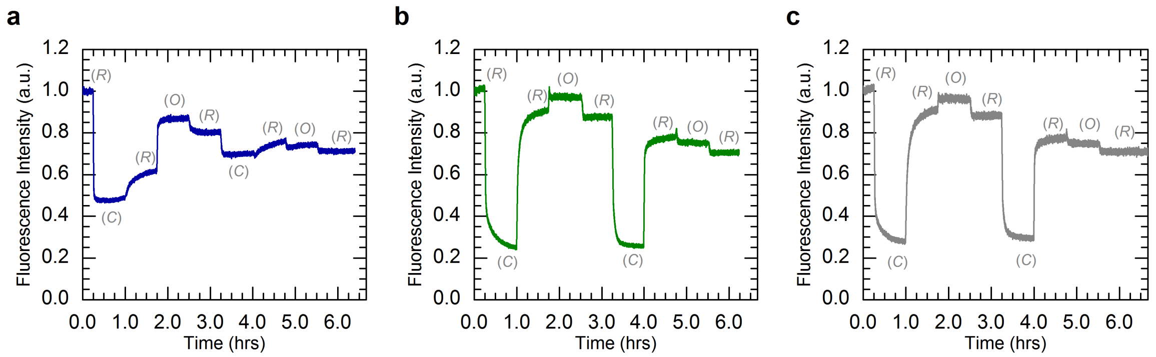

Fig. S6. Kinetics data for operation of the three-state nanomachine at 37 °C in (a) non-heat-inactivated fetal bovine serum, (b) serum from one volunteer, as shown in Fig. 4b, and (c) serum from a second volunteer. The experiment in (a) was performed in 10% FBS/90% PBS, while those in (b) and (c) were performed in 97.5% human serum/2.5% PBS. The fluorescence data were normalized to the initial state intensity. The mechanical states of the nanomachine are Relaxed (R), Closed (C), and Open (O). The data include ~10% fluorescence offsets from excess *A* in solution. The observed overall decreases in fluorescence for the Relaxed states are attributed to dilution from fuel injections.

Supplementary Information S6: Custom Laser-Based Epi-Fluorescence Photometer

To avoid the signal loss inherent in fluorescence measurements in which the excitation and emission are measured at a relative angle of 90° in highly absorbing media, a custom laser-based epi-fluorescence photometer was constructed to measure fluorescence emission from the same surface as the excitation. A schematic of the system is shown in Fig. S7. The system consists of a temperature and current controlled laser diode (Opnext HL6756MG 15 mW continuous wave diode and LXI-GP controller), whose output is modulated by a chopper (Oriel) at 205 Hz. The diode output was tuned to ~670 nm. The drive current and an absorptive neutral density filter were used to adjust the laser intensity, and two mirrors and lenses were used to route and focus the incident beam. A single-edge dichroic mirror (Semrock, long-pass edge at 700 nm, OD2 for wavelengths below 691 nm) reflected the incoming beam toward the sample and allowed the Cy5.5 fluorescence signal to pass through to a photomultiplier module (PMT, Hamamatsu) to amplify the signal. The PMT output was input to a lock-in amplifier (Stanford Research Systems) referenced to the chopper frequency. Because this system was not equipped with a temperature controller for the sample stage, measurements could only be taken at ambient temperature. The baseline detector current of the system was ~6 fA with no solution in the system. A 250 nM sample of the Cy5.5-IBRQ nanomachine in 100% heparinized whole blood yielded a detector current of ~20 pA.

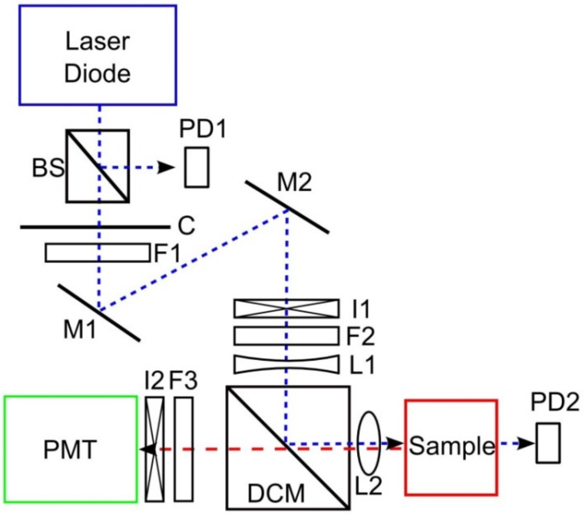

Fig. S7. Schematic of the laser-based epi-fluorescence photometer used to measure nanomachine and linear probe operation kinetics in whole blood. The laser diode signal passes through a beam splitter (BS) allowing a photodiode (PD) to monitor the laser stability. A chopper (C) modulates the beam, which then passes through a neutral density filter (F1) and is guided by mirrors (M1 and M2) through an alignment iris (I1) and bandpass filter (F2). A divergent lens (L1) expands the beam, and the dichroic mirror (DCM) reflects the laser through a focusing lens (L2) onto the sample. Any light transmitted though the sample is recorded by a photodiode (PD2) to ensure system and solution stability. Emitted fluorescence is collected by lens L2 and passes through the dichroic mirror, through a bandpass filter (F3), and through an iris (I2) to a photomultiplier tube (PMT).

Supplementary Information S7: Kinetics and Native PAGE Results for Nicked Nanomachine in Buffer

The sequences for the nicked nanomachine (Fig. S8) are the same as those listed in Table S1, with the *B* strand split into two halves of equal length, B_1 and B_2. The nicked nanomachine was operated in 1×PBS (Fig. S9b), and while the Closed state was still distinct, the Open state fluorescence was not discernable from the Relaxed state. The presence of a nick on the actuator domain allows for a greater range of motion in the Relaxed state, while also decreasing the rigidity of the Open state; hence, the fluorescent distinction between Relaxed and Open states is negligible for the nicked nanomachine. For comparison, operation of the intact nanomachine in 1×PBS is shown in Fig. S9a (same as Fig. 3a).

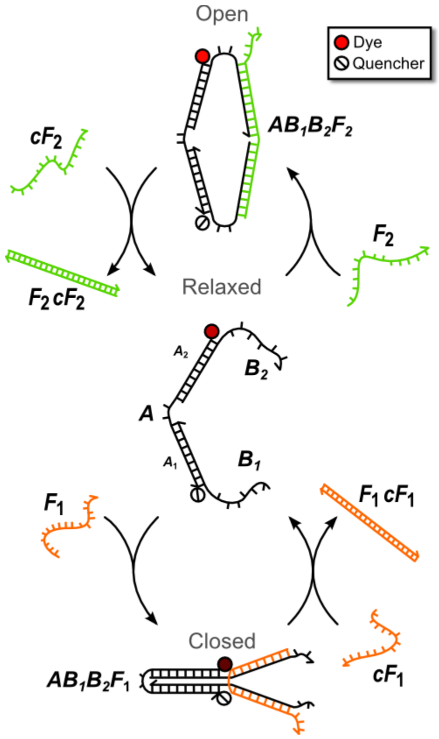

Fig. S8. Schematic of the nicked DNA nanomachine. Similar to the original three-state DNA nanomachine (Fig. 1), the nicked DNA nanomachine transitions between Relaxed, Closed, and Open states with the addition of fuel strands, F_1 and F_2, and their complements, cF_1 and cF_2.

The intact and nicked nanomachines were also compared via native 10% PAGE. Though the migration rates of the Relaxed and Open states were indistinguishable in the presence of a nick on the actuator domain, the migration rate of the Closed nanomachine was decreased when a nick was present. PAGE results from incubation experiments in human serum (Fig. 2) did not indicate this type of degradation occurring for the intact Closed nanomachine; however, the mean lifetimes for the Relaxed and Open nanomachine states reported in this publication may include minimally degraded structures such as the nicked nanomachine, since such structures migrate at the same rate as the intact versions.

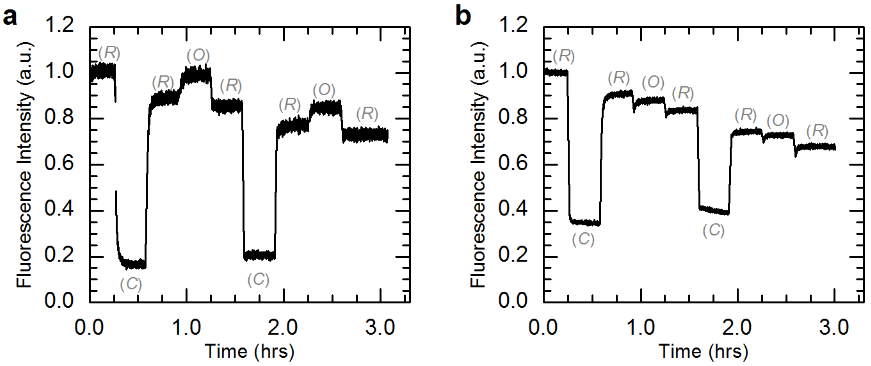

Fig. S9. Kinetics data for operation of the (a) original three-state nanomachine, as shown in Fig. 3, and (b) the nicked nanomachine. Both data sets were collected at 25 °C in 1×PBS. The fluorescence data were normalized to the initial state intensity. The mechanical states of the nanomachine are Relaxed (R), Closed (C), and Open (O). The data include ~10% fluorescence offsets from excess *A* in solution. The observed overall decreases in fluorescence for the Relaxed states are attributed to dilution from fuel injections. As expected, the Closed state of the nicked nanomachine was still distinct, while the transitions from Relaxed to Open states showed drops in fluorescence approximately equal to dilution.

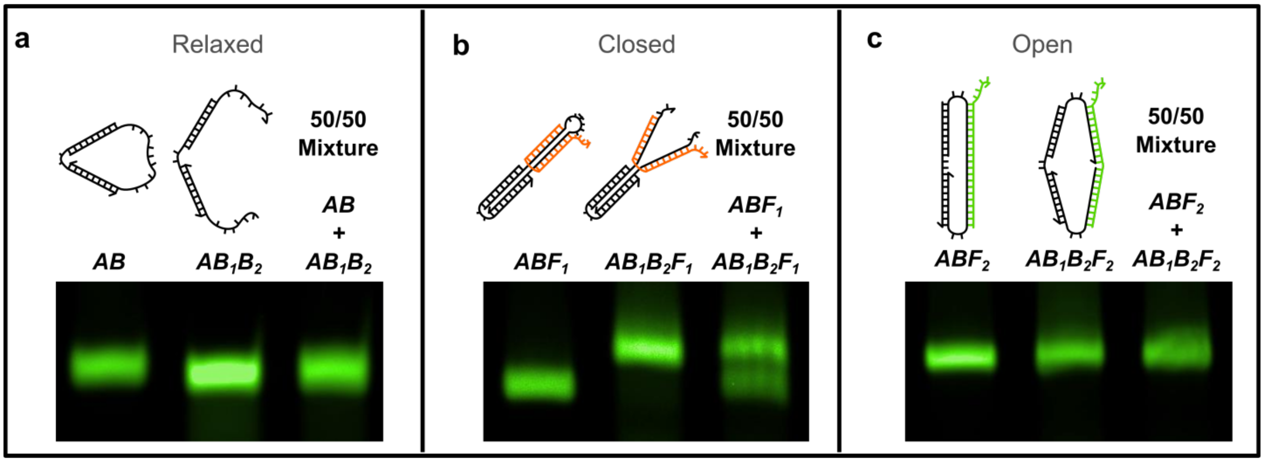

Fig. S10. Native 10% PAGE gel sections for the unlabeled nanomachine in both its original and nicked forms for the (a) Relaxed, (b) Closed, and (c) Open states. A single nick on the nanomachine actuator domain has little discernable effect on the migration rates of the Relaxed and Open states. In the Closed state, the nicked nanomachine migrated significantly slower than its intact counterpart.

References

1. B. G. Moreira, Y. You, M. A. Behlke and R. Owczarzy, *Biochem. Biophys. Res. Commun.*, 2005, **327**, 473-484.
2. W. S. Prince, D. L. Baker, A. H. Dodge, A. E. Ahmed, R. W. Chestnut and D. V. Sinicropi, *Clin. Exp. Immunol.*, 1998, **113**, 289-296.
3. J.-W. Keum and H. Bermudez, *Chem. Commun.*, 2009, 7036-7038.
4. J. W. Conway, C. K. McLaughlin, K. J. Castor and H. Sleiman, *Chem. Commun.*, 2013, **49**, 1172-1174.
